# Sustainable and eco-friendly dyeing of traditional grass cloth with a reactive dye in palm oil medium

**DOI:** 10.1039/d2ra05736k

**Published:** 2022-10-18

**Authors:** Lina Lin, Lexin Xiao, Le Li, Cong Zhang, Md. Nahid Pervez, Vincenzo Naddeo, Youqing Zhang, Md. Shahinoor Islam, Yingjie Cai, Mohammad Mahbubul Hassan

**Affiliations:** Hubei Provincial Engineering Laboratory for Clean Production and High Value Utilization of Bio-based Textile Materials, Wuhan Textile University Wuhan 430200 China yingjiecai@wtu.edu.cn; Hubei Key Laboratory of Biomass Fibers and Eco-Dyeing & Finishing, Wuhan Textile University Wuhan 430200 China; Sanitary Environmental Engineering Division (SEED), Department of Civil Engineering, University of Salerno Fisciano 84084 Italy; Guangzhou Jacky Textile & Technology Co. Ltd Guangzhou 511338 China; Department of Chemical Engineering, Bangladesh University of Engineering and Technology Dhaka 1000 Bangladesh; Fashion, Textiles and Technology Institute (FTTI), University of the Arts London London W1G 0BJ UK mahbubul.hassan@arts.ac.uk

## Abstract

Traditional grass cloth has been used in China for a long time for the manufacturing of various household furnishing textiles and ladieswear. However, traditionally the grass cloth is dyed with reactive dyes in an aqueous medium, but the dyeing process is not sustainable because of high energy and water usage and the production of coloured effluent. In this work, the possibility of palm oil/water dual-phase dyeing of traditional grass cloth with a reactive dye, C.I. Reactive Blue 194 (Reactive Blue 194), was explored. The grass cloth soaked in an alkaline solution with 80–140% pick-up was dyed in a palm oil dyebath containing dye powder dispersed in a palm oil medium. The initial study confirmed that the pre-treatment of the fabric with an alkaline solution with 140% pick-up was beneficial for the uniform distribution of the dye in the fibres. The dyeing process parameters (*e.g.*, fixation temperature, solution pH, and fixation time) for the grass cloth dyeing with the Reactive Blue 194 were optimised by using the Taguchi method. The pH of the alkali pre-treatment solution was found to be the most influential factor, as confirmed by the analysis of variance in terms of the percentage of contribution (94.41%), which was statistically significant (*P* < 0.05). The confirmation tests were carried out under optimal settings, and a higher *K*/*S* (24.06) was found compared with the initial condition (21.51). X-ray diffraction analysis indicated that the dyeing process did not affect the crystallinity of the grass cloth fibres. Furthermore, the recovery of palm oil from the spent dyebath was around 99%, and up to five times recycling and reuse of palm oil were studied for the dyeing of grass cloth. The colour strength of the grass cloths dyed in the palm oil recycled up to five times was similar to the cloth dyed in fresh palm oil. The results show that palm oil can be used as a dyeing medium for the sustainable dyeing of grass cloth with effluent reduction, which can be extended to the dyeing of other textile fibres.

## Abstract 2

Traditional grass cloth has been used in China for a long time for the manufacturing of various household furnishing textiles and ladieswear.

## Graphical abstract

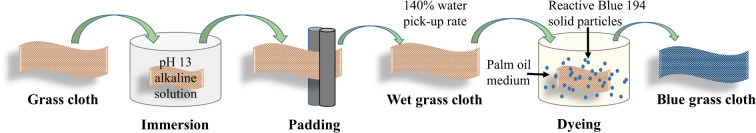

## Introduction

**Fig. 1:**
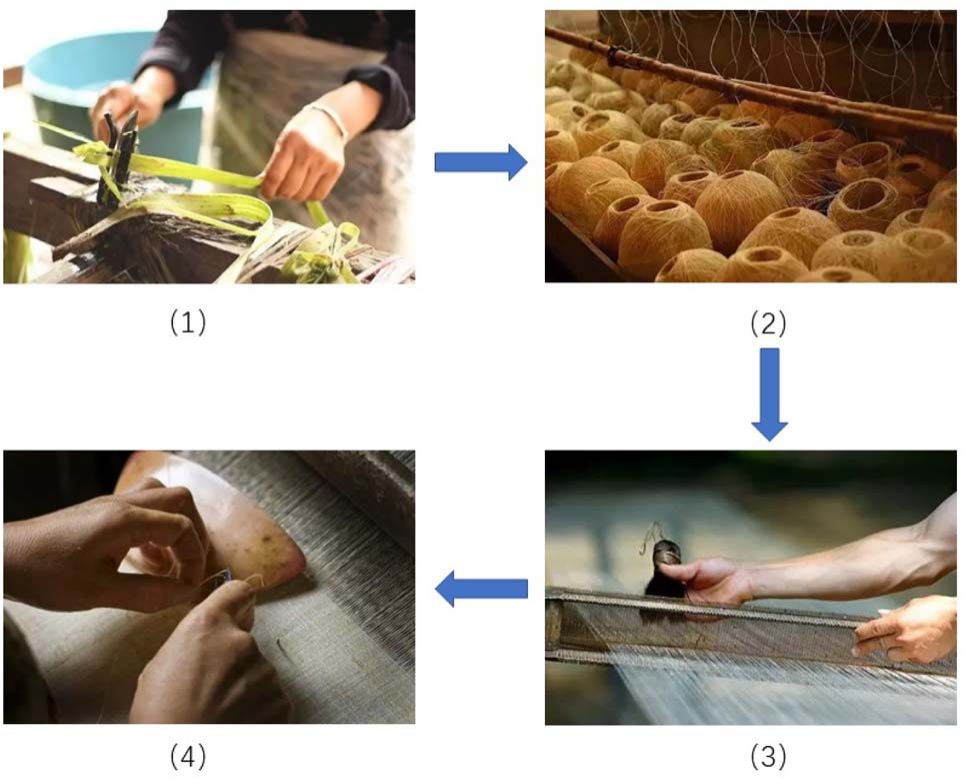
Key process of manufacturing traditional grass cloth: (1) extraction of fibres from ramie phloem, (2) ramie yarns from raw fibres, (3) sizing, and (4) weaving.

In China, the traditional grass cloth, known as Xiabu in Chinese, has been used for more than 6000 years,^1^ and is made from untreated cellulosic fibres containing hemicellulose and lignin extracted from ramie phloem by traditional methods.^2^ Traditional grass cloths are plain-woven fabrics made by cottage industries using handlooms, and they are used in the production of varieties of home furnishing cloths (*e.g.*, wall covering cloths, and tablecloths) and ladieswear. The traditional grass cloth manufacturing process adopted by the Chinese cottage industry is shown in Fig. 1.^3^

Most of the processes, from fibre extraction to spinning of yarn, sizing, and weaving, are carried out by hand. The traditional grass cloth has significance for the textile history of China, and therefore, the National Intangible Cultural Heritage List was established by the Chinese government in 2008 to preserve the old practice of grass cloth making.^4,5^ The colouration of traditional grass cloth to offer colourful products is an alternative option to further develop the culture of traditional grass cloth.

Traditional Chinese grass cloth dyed with reactive dyes in an aqueous medium.^6^ As traditional dyeing uses a large volume of water and also produces coloured effluent, the reduction of chemical and water usage is always important to make grass cloth dyeing sustainable. However, in traditional reactive dyeing, a large quantity of either sodium chloride (NaCl) or sodium sulphate salt (Na_2_SO_4_) is used as an electrolyte to promote dye exhaustion into textile fibres. After dyeing, the salt is difficult to recover from the wastewater.^7,8^ The produced effluent needs to be decolourised for discharging to the local watercourse and for this purpose diverse treatments involving physical, chemical,^9–11^ biological,^12^ and biochemical processes have been studied.^13–18^ However, any kind of decolourisation process is costly and increases the production cost of textiles, and therefore, they are not favourable. To overcome the issue of effluent, it is necessary to develop a cleaner, zero-effluent dyeing process for the dyeing of grass cloths.

Recently, waterless dyeing of cellulosic fibres became a hot research topic, which uses a non-aqueous medium where the solvent can be recycled and reused for successive dyeing. Various solvents, such as liquid ammonia,^19–23^ decamethylcyclopentasiloxane or D5,^24–28^ cottonseed oil,^29^ and supercritical carbon dioxide^30^ as an alternative to the aqueous medium. In liquid ammonia dyeing, the colouration and mercerisation of cellulosic fibres are achieved simultaneously. However, the need for non-standard dyeing equipment and the high dyeing cost made it not feasible for industrial production. In the D5-based dyeing process, high exhaustion and fixation of reactive dye can be achieved, and D5 can be recycled and reused. However, it is impossible to recycle 100% of the D5, some of which remains in the dyed textiles, and the discharge of D5 into the environment has potential environmental issues.^31^ In addition, OEKO-TEX STANDARD 100 stipulated that the concentration of D5 in the dyed fabrics should be lower than 0.1% (wt%), which is challenging for the industrialisation of D5-based dyeing technology. Cottonseed oil also can be used as a dyeing medium as an alternative to the aqueous medium, which can be recycled and reused, eliminating the production of effluent. However, the conversion of some unsaturated fatty acids to saturated fatty acids after reactive dyeing, especially at a high pH, is troublesome in recycling and reusing cottonseed oil.^32^

Palm oil has high heat and oxidative stabilities^33^ and could be an alternative to cottonseed oil for effluent-free dyeing but is rarely considered for this purpose. It has a few unsaturated fatty acids and, therefore, less chance of conversion to saturated fatty acids during dyeing, and it is considerably cheaper and easier to recycle than cottonseed oil. Unlike D5, the release of residual palm oil from the dyed cloth to the washing effluent in the post-dyeing soap-washing is not an issue as it is not harmful to the environment because of its biodegradability. Followingly, there is a possibility that the chemical potential of the dyes in the palm oil dyeing medium might be significantly boosted, which would result in an increase in dye fixing and a reduction in discharges. Besides, it is realized that there would be a reduction in the amount of energy required to attain the same temperature throughout the dying process since palm oil has less heat capacity than water.^34,35^ Therefore, in this article, we are reporting the colouration of traditional grass cloth with Reactive Blue 194 dye in a palm oil medium for the first time. An L^9^ orthogonal experimental scheme and *K*/*S* values were used to optimize dyeing conditions.

## Experimental

### Materials and reagents

**Fig. 2:**
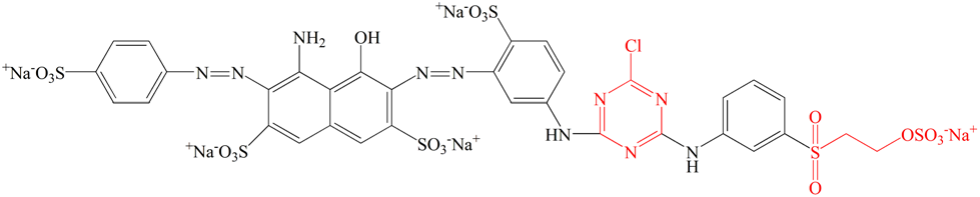
Chemical structure of C.I. Reactive Blue 194.

The traditional grass cloth (without degumming) was purchased from the local market. Commercial-grade C.I. Reactive Blue 194 was bought from Shanghai Jiaying Chemical Company (China). The molecular structure of the dye used in this work is displayed in Fig. 2. Palm oil (food-grade) was purchased from Liu'an Shi Dongsheng Youzhi Xiaoshou Company (China). Nonionic detergent (Luton 500) was supplied by Dalton UK Company (China). Sodium carbonate (99.5%) and sodium hydroxide (>98%) were purchased from Shanghai Aladdin Biochemical Technology Co., Ltd.

### Dyeing of traditional grass cloth

In the traditional aqueous dyeing of grass cloth, it is directly dyed without scouring pretreatment, to maintain the gummy materials in the cloth. Therefore, in this work, the grass cloth was dyed in palm oil without scouring pretreatment. For dyeing using palm oil as a medium, the cloth was first immersed in alkaline aqueous solutions of pH 11 to 13 and squeezed by padding rollers with 60–140% pick-up. For pH below 12, Na_2_CO_3_ was used, but for pH higher than 12, NaOH was used to adjust the pH. The wet grass cloth was immersed in a palm oil dyebath containing 3% o.m.f (on the mass of fabric) Reactive Blue 194 dye evenly dispersed and the dyeing was carried out using a material to liquor ratio of 1 : 20. The dyeing parameters (Table 1) were selected to design an L^9^ orthogonal experimental scheme to determine the optimum dyeing conditions.

**Table 1:** Parameters of grass cloth dyeing for the L^9^ orthogonal experimental scheme

Fixation temperature (°C)	pH of alkaline solution	Fixation time (min)
60–80	11–13	30–50

**Fig. 3:**
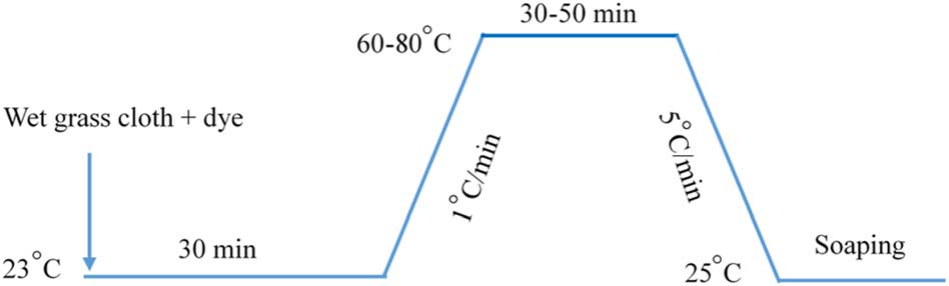
Dyeing process of grass cloth with Reactive Blue 194 in palm oil.

An infrared radiation heated laboratory-dyeing machine was used for all dyeing works (Automatic Prototype, Model: A-12, AQUA, China), and the dyeing of grass cloth was carried out according to the process shown in Fig. 3. After dyeing, oils were separated from the dyed samples by centrifuging them for 10 s and then dried in an oven at 60 °C for 15 min followed by washing in a soap solution containing 2 g L^−1^ non-ionic detergent at material to liquor ratio of 1 : 50.

### Characterisation of dyed grass cloth samples

The colour strength (*K*/*S*) of dyed cloth samples was measured at 20 random places using a reflectance spectrophotometer (CHN-Spec CS-650A, Hangzhou Color Spectrum Technology Company, China). The colour strength was measured at the wavelength of maximum absorption of the dye and the average colour strength is reported here. The X-ray diffraction (XRD) analysis of the original and dyed grass clothes was conducted using an X-ray diffractometer (Rigaku Ultima III, Tokyo, Japan) with a 2*θ* angle range from 5 to 55° with 0.02° per step. The XRD pattern was analysed using the FitYK 1.3.1 program for the deconvolution of the peaks. Then, the detected areas of the crystalline (*I*_c_) and amorphous (*I*_a_) regions were used to calculate the crystalline index (CI) using eqn (1). For measuring the dye exhaustion percentage (*E*%) by eqn (2), the absorbance of dye solution before dyeing (*A*_0_) and after dyeing (*A*_1_) was measured with a Cary 100 UV-vis spectrophotometer (Agilent, USA). The absorbance values of the washing effluent (*A*_2_) were measured to calculate the dye fixation efficiency (*F*%) and the total dye fixation rate (*T*%) using eqn (2)–(4). The colourfastness to washing and rubbing was measured according to the test methods of ISO 105-C06:2010 and ISO105-X12:2011, respectively. The tensile strength of the grass cloth was measured based on the GB/T 3923.1-1997 test method with an electronic strength tester (Laizhou Electron Instrument Company, China) for fabric. The stiffness of the grass cloth was measured according to the GB/T18318.1-2009 test method. The wettability of the grass cloth was characterised by its capillary effect, which was detected according to the FZ/T01071-2008 test method.1
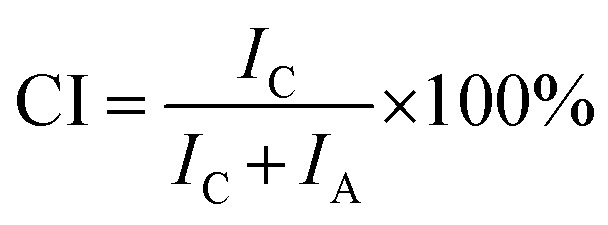
2
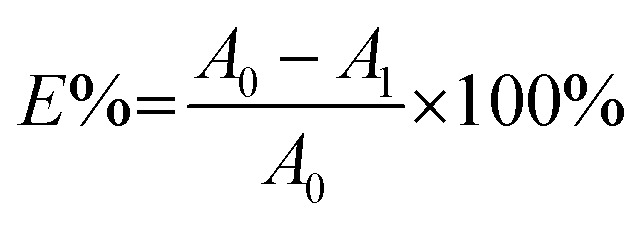
3
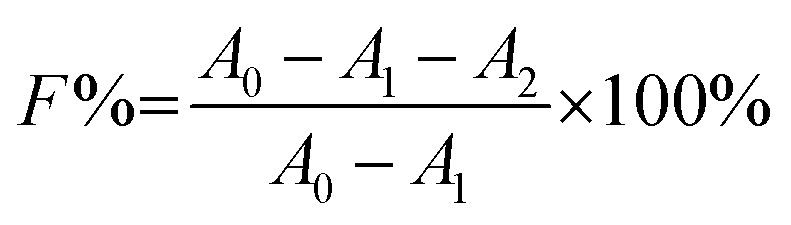

4 *T*% = *E*% × *F*% × 100

### Recycling palm oil from residual dyebath

After dyeing, extra water (5% of residual dyebath) was added to the residual dyebath along with vigorous agitation for 1 min to dissolve the residual dyes, followed by separation of palm oil by centrifugation, and the water from the oil was separated by decanting. Finally, the separated clean palm oil was collected and used for the successive dyeing.

## Results and discussion

### Influence of alkaline solution pick-up rate on colour uniformity

**Fig. 4:**
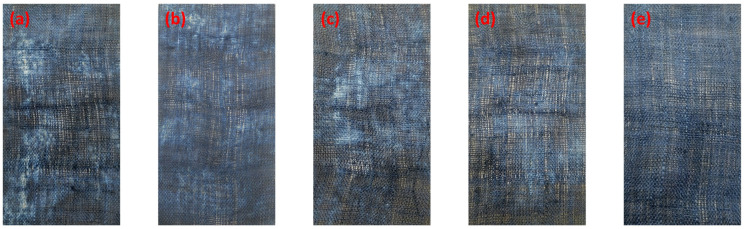
Optical images of dyed grass cloth samples pre-treated with various aqueous alkali solution pick-up: (a) 60%, (b) 80%, (c) 100%, (d) 120%, and (e) 140%.

**Fig. 5:**
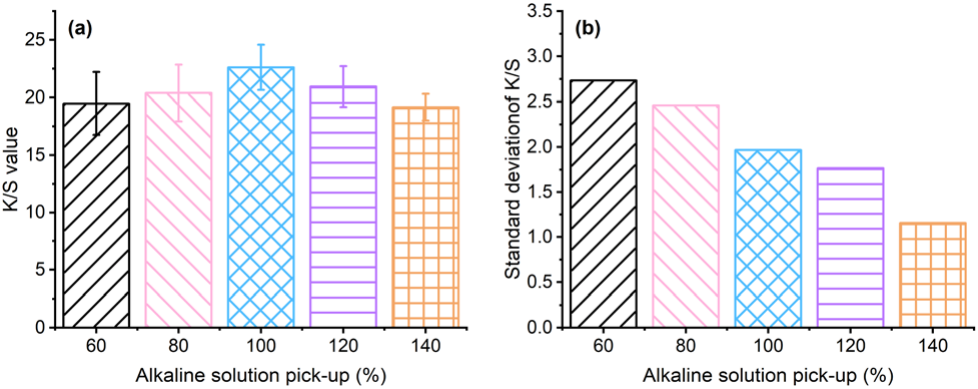
(a) *K*/*S* values of grass cloths dyed with Reactive Blue 194 with an aqueous alkaline solution pre-treatment with a pick-up of 60–140% and (b) the relative standard deviation of the *K*/*S* values.

The optical image of grass cloth dyed with Reactive Blue 194 pre-treated at pH 13 with various pick-up percentages is shown in Fig. 4, and the *K*/*S* values are shown in Fig. 5. The *K*/*S* of the dyed samples increased with an increase in the alkaline solution pick-up percentage and increased to 22.6 at a pick-up of 100% (Fig. 5a), indicating that the dye exhaustion efficiency was the highest for the 100% alkaline solution pick-up as for the lower alkaline solution pick-up rate, the wetting of the grass cloth was incomplete, resulting in low adsorption of dye. The grass cloth was wetted completely when the alkaline solution pick-up was 100%. The *K*/*S* value decreased with a further increase in the alkaline solution pick-up, as the excess water was transferred from the fabric to the palm oil dyebath. The dye dissolved in the water transferred to the palm oil dyebath was not absorbed into the fabric and therefore, the dye exhaustion decreased when the alkali solution pick-up was more than 100%.

Possibly, some of the dye adsorbed into the wet grass cloth was desorbed into the water in the dyebath. The colour uniformity of dyed fibres is a primary factor for dyeing, which is characterised by the standard deviation of its *K*/*S* values. The lower standard deviation refers to better colour uniformity. Although the highest *K*/*S* of the grass cloth was associated with the alkaline solution pick-up rate of 100%, the standard deviation of *K*/*S* values decreased with an increase in the alkaline solution pick-up rate, which was from 2.73 for the alkaline solution pick-up rate of 60% to 1.15 for the alkaline solution pick-up rate of 140% (Fig. 5b). In other words, the water content in the grass cloth was beneficial for the uniform distribution of dye in the fibres. The rough and diversified chemical components of raw ramie yarn and the lumpy surface of the grass cloth inevitably resulted in a slightly high standard deviation compared with cotton woven fabric.^36^ The colour shade of dyed samples produced uniform shades when the alkaline solution pick-up was 140%. Considering the colour strength and uniformity factors, the alkaline solution pick-up of 140% was selected for all dyeings of the grass cloth.

### Orthogonal experimental dyeings of the grass cloth with reactive dyes

The dyeing of the grass cloth based on the L^9^ orthogonal experimental scheme was implemented, followed by the soap-washing process. The *K*/*S* values of the dyed samples are listed in Table 2. Normally, the higher *K*/*S* of the dyed sample indicates the higher dye concentration in fibres.^37^ The highest *K*/*S* value of the dyed sample was 21.51, which was achieved for the fabric sample treated and pH 13 and then dyed at the fixation temperature of 80 °C, and fixation time of 40 min (sample 9 in Table 2).

**Table 2:** *K*/*S* values of the dyed grass cloth samples by the L^9^ orthogonal experimental scheme

No.	Fixation temperature (°C)	pH of alkaline solution	Fixation time (min)	*K*/*S*
1	60	11	30	5.94
2	60	12	40	14.67
3	60	13	50	18.80
4	70	11	40	7.95
5	70	12	50	18.22
6	70	13	30	20.54
7	80	11	50	6.44
8	80	12	30	12.89
9	80	13	40	21.51

### Taguchi analysis of *K*/*S*

#### S/N ratio assessment

**Fig. 6:**
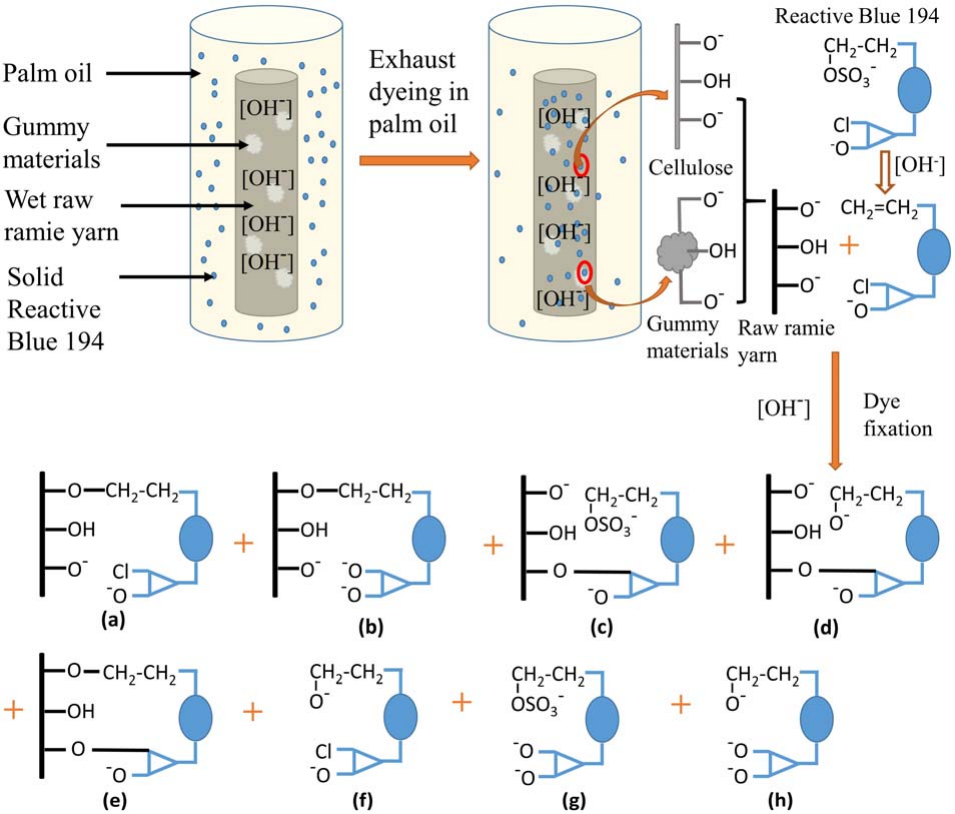
Mechanism of dye adsorption and fixation of Reactive Blue 194 with grass cloth in a palm oil medium. (a) The fixed dye with an MCT group, (b) the fixed dye with a hydrolysed MCT group, (c) the fixed dye with a VS group, (d) the fixed dye with a hydrolysed VS group, (e) the dye fixation with two covalent bonds, (f) the unfixed dye with a hydrolysed VS group, (g) the unfixed dye with a hydrolysed MCT group, and (h) the unfixed dye with two hydrolysed groups.

**Fig. 7:**
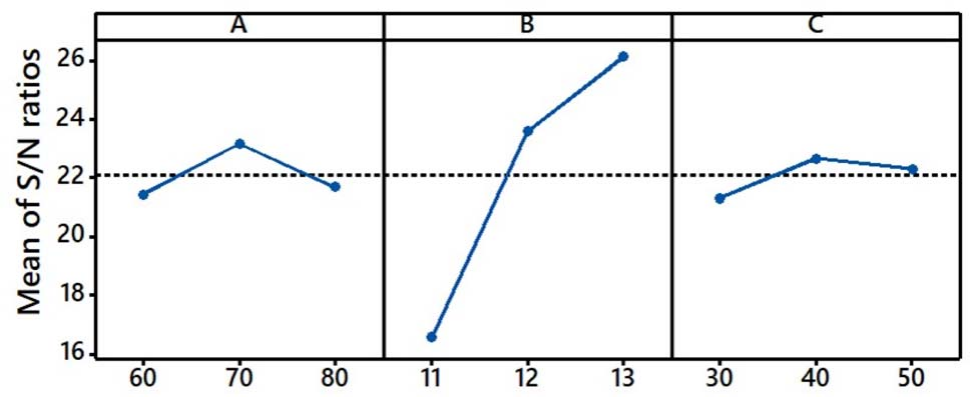
Main effect plot for S/N ratios of the *K*/*S* value.

The influencing order of the three variables on the colour strength (*K*/*S*) of dyed samples in the set condition ranges is shown in Table 3, which was in the order of: the pH of alkaline solution > fixation temperature > fixation time. The *K*/*S* value of the dyed sample was dependent on dye exhaustion and dye fixation.^38,39^ During the dyeing, the dye powder was suspended evenly in the palm oil medium and tended to be adsorbed into the wet grass cloth owing to its hydrophilic property. After adsorption, the adsorbed dye was prevented from transferring to the hydrophobic palm oil because of its insolubility in palm oil, resulting in high exhaustion. In other words, the dye exhaustion process was irreversible. The mechanism of dye adsorption and fixation is shown in Fig. 6. At the high pH of the solution (pH 11–13), part of the hydroxyl groups (–OH) of raw ramie yarn was ionized to anionic groups (–O^−^), and the reactive groups of Reactive Blue 194 were active, therefore, covalent bonds were formed between the raw ramie yarn and reactive dye.^40^ Because Reactive Blue 194 is a bifunctional reactive dye having monochlorotriazinyl (MCT) and sulphatoethylsulphone (VS) reactive groups. At alkaline conditions, the vinyl sulphone sulfate group (–CH_2_CH_2_OSO_3_^−^) transferred to vinyl sulphone group (–CH_2_=CH_2_), which is reactive in alkaline conditions. Therefore, Reactive Blue 194 could form a covalent bond *via* VS (Fig. 6a and b) or MCT group (Fig. 6c and d) or double covalent bonds *via* MCT and VS groups (Fig. 6e) with raw ramie yarn. In addition, at alkaline conditions, MCT and VS reactive groups were probably subjected to hydrolysed simultaneously and lost their reactive property (Fig. 6b, d and f–h). Therefore, the fixation temperature was not considered to affect the dye exhaustion. Meanwhile, as depicted in Fig. 7a, *K*/*S* values showed a rise–fall tendency with little distinction in the fixation temperature range, indicating lowered sensitivity.

**Table 3:** Response table for S/N ratios in *K*/*S* values of dyed grass cloths

Symbol	*A*	*B*	*C*
Level	Fixation temperature	pH of alkaline solution	Fixation time
1	21.43	16.55	21.31
2	23.16	23.58	22.66
3	21.68	26.13	22.29
Delta	1.73	9.58	1.35
Rank	2	1	3

During the dyeing, with an increase in dyeing temperature to the target fixation temperature and dyeing time extension, and under strongly alkaline conditions, the fibre of the grass cloth swelled well, which was beneficial for dye migration from the surface to the interior of fibres.^41^ Meanwhile, the strong alkaline conditions were effective not only in swelling gummy materials but also in acting on the sites of cellulosic fibres, gummy materials, and reactive groups of the reactive dye. These benefits and a suitable fixation temperature promoted the covalent bond formation between the dye and the grass cloth, as shown in Fig. 6. In addition, Fig. 7b shows that the *K*/*S* increased with an increase in the pH from 11 to 13, indicating that the *K*/*S* difference among the dyed samples was enlarged, increasing the pH factor sensitivity of *K*/*S* values. Reactive Blue 194 is commonly applied at 60°–80 °C for cellulosic fibre dyeing. The activities of MCT and VS reactive groups are different; the former requires strongly alkaline conditions, while the latter requires less alkaline conditions for the reaction. Thus, the pH range covered the requirements of MCT and VS reactive groups and was beneficial for dye fixation.^42^ Therefore, the promotion of dye fixation increased the *K*/*S* value of the dyed sample because the unfixed reactive dyes were washed off in the subsequent soaping process. In summary, the pH of the pre-treating alkaline solutions from pH 11 to 13 was more sensitive in dye fixation than the fixation temperature, indicating that the pH of the alkaline solution was more sensitive to achieving a higher *K*/*S* value for the dyed samples.

During the dyeing, the solid dye in palm oil was adsorbed quickly. Thus, the influence of the fixation time factor on dye adsorption was ignorable. In other words, the fixation time factor responded only to the dye fixation. As shown in Fig. 7c, the *K*/*S* change with an increase in fixation time from 30 to 50 min exhibited a rise–fall tendency, which was similar to the change in the fixation temperature factor. However, the *K*/*S* distinction among the dyed samples was slight compared with the fixation temperature factor. Therefore, the fixation time was the least sensitive factor in the *K*/*S* of the dyed sample in the dyeing process.

### Interaction plots

**Fig. 8:**
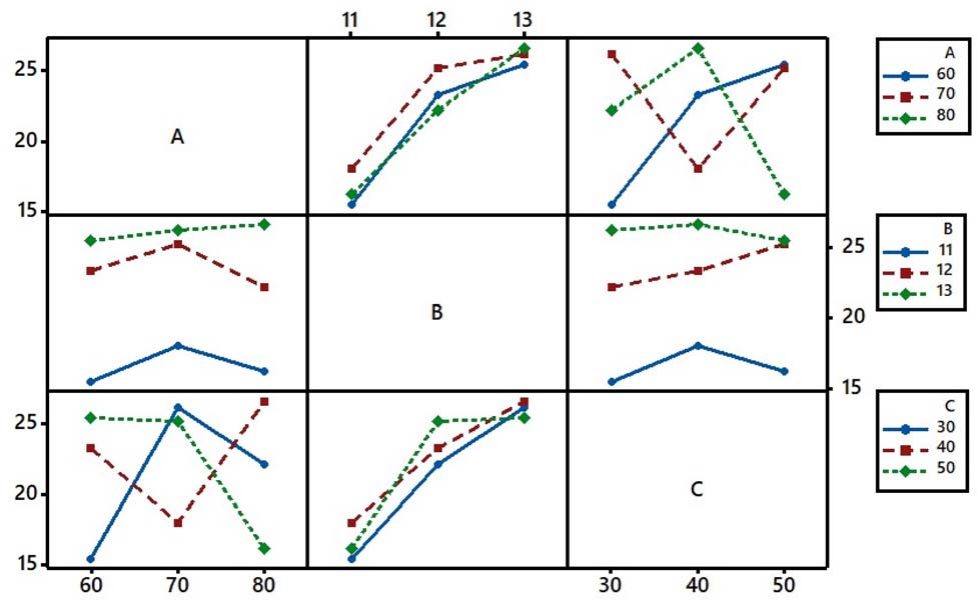
Full interaction plot for S/N ratios (*T*%).

The *K*/*S* of the dyed sample was influenced by the fixation temperature, pH of the aqueous alkaline solution used for the pre-treatment, and dye fixation time. Hence, the interaction behaviour of these factors between the levels needed to be understood, which is shown in Fig. 8. It showed a substantial interaction between the fixation temperature (*A*) and fixation time (*C*). Parallel lines in the interaction between *A* and *B* or *B* and *C* indicated that their levels were interdependent.^43,44^ However, the increase in pH increased the interaction behaviours between *A* and *B* at 80 °C and between *B* and *C* for the 50 min fixation time.

### Analysis of variance

Analysis of variance (ANOVA) was applied to determine the relative importance of factors in an orthogonal experimental scheme.^45^ The most important value obtained from ANOVA was the *P* value; values higher than 0.05 meant that the variable was significant on the influence of the analysed target. Otherwise, it was insignificant.^25,46^ The results of ANOVA for the S/N ratio of the *K*/*S* are listed in Table 4. Among the three variables, only the factor of the pH of the alkaline solution was significant because its *p*-value was 0.004 (<0.05), and its contribution percentage (*P*%) was up to 94.41%. The other two variables were insignificant and had low contribution percentages. The ANVOA findings corroborated the ranked order in Table 3.

**Table 4:** ANOVA for S/N ratios of *K*/*S*

Source	DF	SS	MS	*F*	*p*-Value	Remarks	*P* (%)
*A*	2	5.23	2.616	9.04	0.100	Not significant	3.35
*B*	2	147.58	73.789	255.10	0.004	Significant	94.41
*C*	2	2.93	1.463	5.06	0.165	Not significant	1.87
Residual error	2	0.58	0.289				0.37
Total	8	156.32					

### Analysis of residual plots

**Fig. 9:**
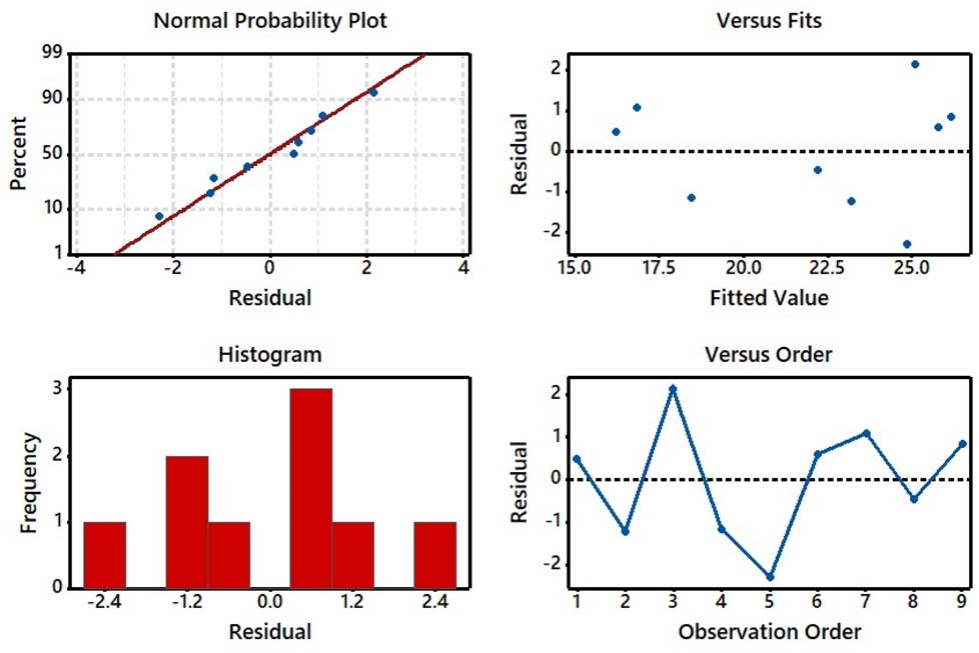
Residual plots for S/N ratios on normal probability, *versus* fits, histogram, and *versus* order.

The residual plots for the *K*/*S* of the dyed sample were investigated (Fig. 9) in which normal probability plot, histogram, and *versus* fits and orders were considered leading directories. The residual plots might be used to assess the model's suitability and compliance with the analysis assumptions.^47,48^ As shown in the normal probability plot (top right side), a majority of spots were on or near the line, which meant that the residuals were distributed normally in the dyeing process. In the histogram (bottom left side), a few observations were counted with variance points and a less skewed pattern. The residual *versus* fitted (top right side) was used to determine whether the output results were impacted by the set parameters. The position of points was unevenly oriented; particularly, lower points were more inclined horizontally, affirming the transformation of the residual response variable. According to residuals *versus* order (bottom right side), observed residuals were nearly placed on each other, indicating that residuals were strongly correlated and dependent on the dyeing procedure.^36^

### Fitted plot assessment

**Fig. 10:**
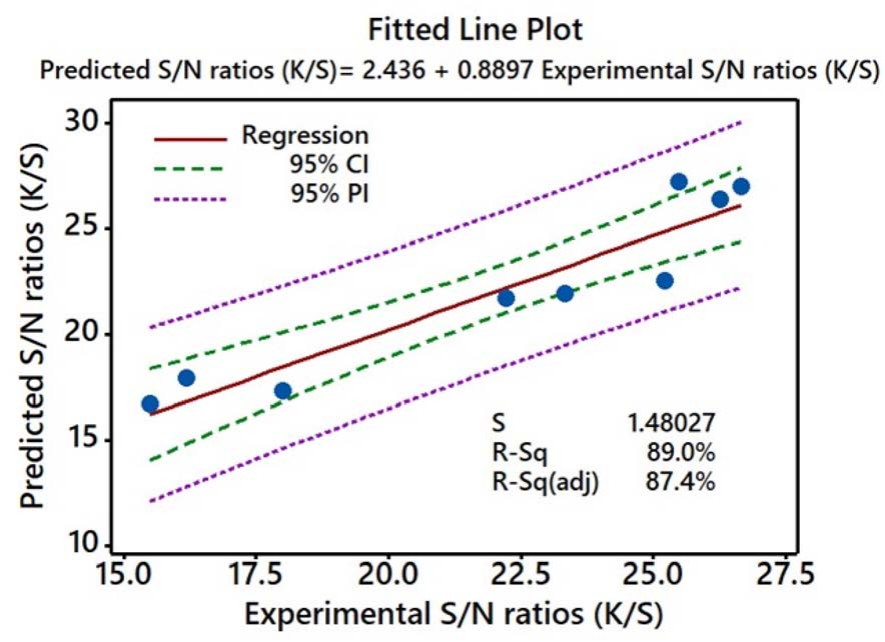
Fitted lines for the experimental S/N ratios (*K*/*S*) and predicted S/N ratios (*K*/*S*).

A fitted plot assessment delivers the regression equation, which allows accurate computation and comparison of predicted values depending on experimental conditions.^49,50^Fig. 10 displays the fitted graph of predicted *versus* experimental responses for the *K*/*S* values of the dyed samples. The data implied a good match for both the experimental and predicted *K*/*S* values, as the difference between *R*^2^ (89.0%) and adjusted *R*^2^ (87.4%) was minimal. In this way, they were all in complete accord with one another. With a difference of 1.6, the ability of the model to predict the response accurately and within a reasonable margin of error is shown. Along with this, a Pearson correlation coefficient of 0.889 between predicted *K*/*S* values and experimental *K*/*S* values was found. As shown by the *P* value of 0.000 for this correlation coefficient, the predicted and actual *K*/*S* values were highly correlated.^50^

### Confirmation test

Confirmation testing is a need in Taguchi techniques and is highly suggested when using statistical methodologies to help explain the acquired results. It was the primary purpose of this study to determine whether the tests and replies were legitimate.^51^Table 5 displays the results of the confirmation trials. Next, whether the process was running at its most efficient level needed to be ensured. Using the software, we were able to predict the results that could be achieved. As a consequence, the experiment was carried out using the ideal parameters. It was found that the S/N ratio had improved a lot (with an increase in the S/N ratio of 0.97).

**Table 5:** Results of the confirmation experiment

Conditions	Initial parameters	Prediction	Confirmation experiment
Level	A3B3C2	A2B3C2	A2B3C2
*K*/*S*	21.51	22.35	24.06
S/N	26.65	27.77	27.62
Improvement in the S/N ratio		0.97	

The predicted *K*/*S* value of the sample dyed at 70 °C and pH 13 for 40 min was 22.35, and the *K*/*S* value of the dyed sample from the confirmation experiment was 24.06. This result indicated that the dyeing parameters obtained from the Taguchi analysis were further optimised. Compared with the initial dyeing, the confirmation test not only increased the colour strength of the dyed grass cloth from 21.51 to 24.06 but also decreased the fixation temperature from 80 °C to 70 °C. It lowers the 10 °C requirement for the 40 min fixation time, *i.e.*, reducing heating energy consumption for dyeing.

### Characterisation

The *E*%, *F*%, and *T*% values of the grass cloth dyeing under the optimised conditions in palm oil media are displayed in Table 6. It was obvious that the *E*% was 95.41% in the palm oil dyeing because of the high affinity of Reactive Blue 194 dye towards the wet grass cloth and the one-direction adsorption procedure, that is, irreversible adsorption. However, 50.61% of *F*% resulted in only 48.30% of *T*%. The colourfastness to washing and rubbing of the dyed grass cloth was excellent, and all of them were higher than 4.

**Table 6:** Dyeing performance of Reactive Blue 194 dyed grass cloth in palm oil

Dyeing			Wash fastness		Rubbing fastness	
*E*% (%)	*F*% (%)	*T*% (%)	Staining	Fading	Dry	Wet
95.41 ± 1.03	50.61 ± 0.99	48.30 ± 1.45	4–5	5	4–5	4

**Fig. 11:**
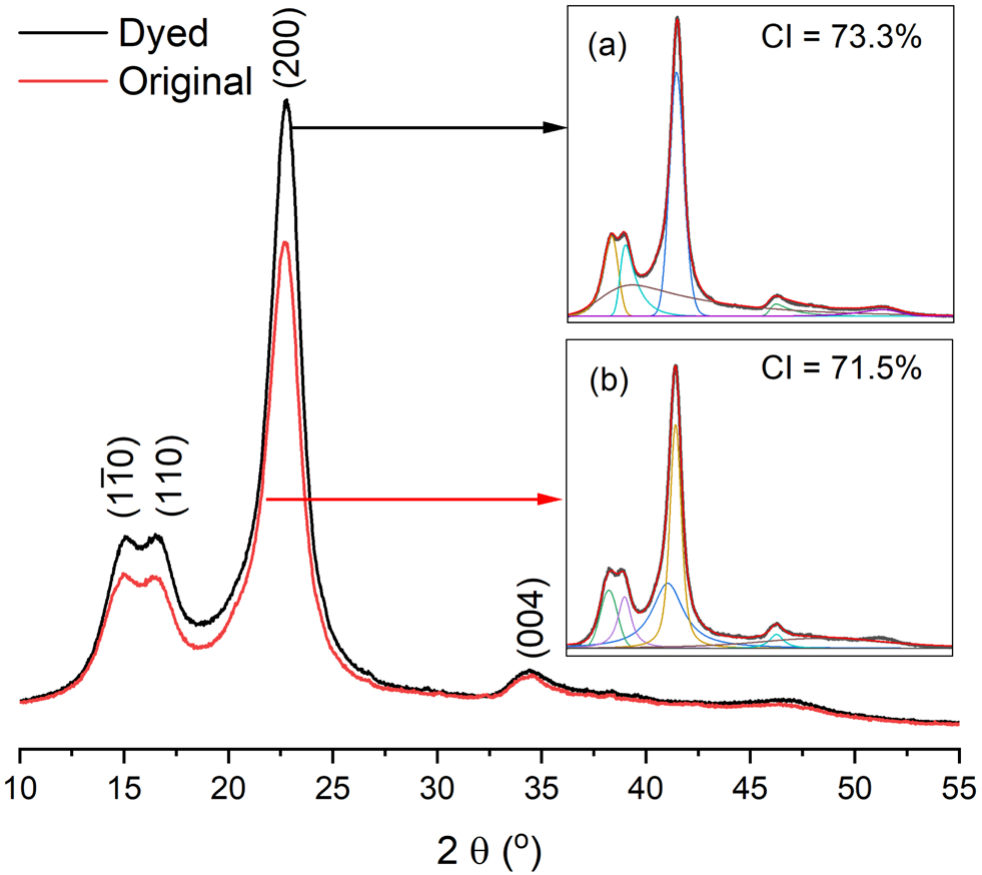
XRD patterns of (a) dyed and (b) original grass clothes.

The gummy materials did not have a crystalline region; hence, all crystalline regions were ascribed to ramie cellulosic fibres.^6^ The XRD patterns of the original grass cloth and the grass cloth dyed in palm oil media under the optimised conditions are shown in Fig. 11. The two theta values were at 15.1°, 16.6°, 22.8°, and 34.4°, corresponding to the (11̄0), (110), (200), and (004) lattice planes, respectively, of the cellulose I pattern in both XRD curves.^52^ The two XRD patterns were almost identical, and the CI values of both compounds fell within the range of each other (71.5% and 73.3% for the original and dyed grass clothes, respectively), suggesting that the dyeing process did not affect the crystallinity of the grass cloth.^53,54^

The mechanical properties of the grass cloth before and after dyeing with Reactive Blue 194 dyed grass cloth in palm oil under optimum conditions were measured and the results are presented in Table 7. The breaking forces of the dyed sample in the warp and weft directions decreased to 382.43 N from 535.54 N and 372.39 N from 464.25 N, respectively. In addition, the elongation of the grass cloth increased from 4.36% to 9.70% and from 5.55% to 8.72% in the warp and weft directions respectively. The bending length and flexural rigidity reduced to 14.17 cm and 430.67 mN cm^−1^, reflecting a decrease of 17.2% and 43.3%, respectively. The wicking height used to characterize the hygroscopicity of the grass cloth increased in warp and weft directions to 6.34 cm from 6.16 cm and 6.82 cm from 5.66 cm, respectively. The hygroscopicity in the weft direction was higher than the warp direction due to the low twist in yarn in the weft direction than the warp direction. The fibres in the warp yarns were more loosely bundled than the yarn in the warp direction allowing faster wetting of the yarns in the fabric. In addition, during dyeing in palm oil, some impurities from the grass cloth were potentially dissolved in palm oil, contributing to the changes in the mechanical properties.

**Table 7:** Mechanical properties of the original and dyed grass clothes

Item		Original	Dyed
**Tensile strength**			
Breaking forces (N)	Warp direction	535.54	382.43
	Weft direction	464.25	372.39
Elongation (%)	Warp direction	4.36	9.70
	Weft direction	5.55	8.72

**Stiffness**			
Bending length (cm)		17.12	14.17
Flexural rigidity (mN cm^−1^)		759.54	430.67
Wicking height (cm)	Warp direction	6.16	6.34
	Weft direction	5.66	6.82

### Recycling palm oil for dyeing

In the recycling of palm oil from the residual dyebath, a small amount of water was added to the residual dyebath to dissolve the soluble materials such as solid dye and wet the short fibres that dropped out from the grass cloth during dyeing; the water addition was beneficial for the purification of palm oil. Therefore, palm oil was easily recycled from water, and its recycling efficiency was around 99%. The recycled palm oil was used for the dyeing under optimum conditions. The colour strength and CIE L*a*b* values of grass cloth samples dyed in palm oil medium up to five successive recycle and reuse are shown in Table 8. Compared to the fresh palm oil dyeing, the colour strength and L* value of the dyed samples with recycled palm oil were similar, indicating that the dyeing process did not affect the quality of palm oil. In addition, the chromatic values exhibited a high reproducibility for preparing the dyed grass cloth.

**Table 8:** Chromatic values of dyed samples with consecutive recycled palm oil

	Fresh	Recycle 1	Recycle 2	Recycle 3	Recycle 4	Recycle 5
*K*/*S*	24.06	23.90	24.32	24.45	24.02	23.89
L*	18.23	18.31	18.86	17.56	17.97	18.42
a*	−0.84	−0.75	−1.05	−0.86	−0.71	−0.95
b*	−7.58	−7.63	−8.24	−7.92	−6.99	−7.74

### Evaluation of environmental impacts

The chemical consumption for grass cloth dyeing in palm oil was compared with the cotton dyeing in water media. The findings are listed in Table 9. A liquor ratio of 1 : 20 was applied for the dyeing of the grass cloth in the palm oil medium, while a ratio of 1 : 6–1 : 10 was used for the aqueous dyeing of cotton fabric samples. After dyeing, 99% of palm oil was possible to recycle, while water recycling was 70–80% as some water was lost due to evaporation. Before immersing in the palm oil dyebath, the grass cloth was wet with a water pick-up of 140%, and a small amount of water was added to the residual palm oil dyebath, which was 5% of the residual palm oil dyebath. Although the *E*% was high up to 96% after dyeing, around 50% of *T*% reduced the dye usage efficiency. In contrast, in aqueous dyeing, approximately 20–50% (ref. ^55^ and 56) of the applied reactive dye becomes hydrolysed and does not fix with the fibres. The advantage of this palm oil dyeing was that it was salt free, which is needed abundantly in the aqueous reactive dyeing at the level of 40–100 g L^−1^. It was difficult to treat electrolytes in wastewater, and a huge amount of salt was discharged into the environment, causing pollution. Alkali treatment is essential for dye fixation in reactive dyeing. In aqueous cotton dyeing, 10–40 g L^−1^ of NaOH/Na_2_CO_3_ is needed for efficient dye fixation, while in palm oil medium dyeing, only 5 g L^−1^ of NaOH is required.^29^ The water used in the dyebath for cotton dyeing was 6–10 times the dyeing fabric weight (F.W.), but it was only 1.4 times of the grass cloth in palm oil dyeing. Therefore, alkali consumption was dramatically reduced in palm oil dyeing. Generally, the dyeing of the grass cloth in palm oil media was a cleaner dyeing pattern.

**Table 9:** Chemical consumption and waste produced

Item	Palm oil dyeing	Conventional water dyeing
Palm oil (L)	F.W. × 20	0
Water (g)	F.W. × 1.4 + F.W. × 20 × 0.05	F.W. × (6–10)
Dye waste (%)	52	20–50
Electrolytes (g)	0	F.W. × (6–10) × (40–100)
Alkali (g)	F.W. × 1.4 × 5	F.W. × (6–10) × (10–40)
Recycle dyeing media (%)	99	70–80

## Conclusions

Considering the current environmental issues of textile dyeing, sustainable and eco-friendly dyeing of the traditional grass cloth with Reactive Blue 194 in a palm oil medium was established in the present study. The application of the Taguchi design assisted in achieving the optimal dyeing conditions (A2B3C2), which included a fixation temperature of 70 °C, pre-treatment with an alkaline solution of pH 13, and a fixation time of 40 min. Particularly, Taguchi's design also allowed the development of an energy-efficient dyeing operation with a much higher *K*/*S* value, such as the initial fixation temperature of 80 °C and *K*/*S* of 21.51. However, after the optimum conditions were obtained from the S/N ratio analysis, the fixation temperature was reduced to 70 °C, and the *KS* value was 24.06 (improvement in the S/N ratio was 0.97). The results of ANOVA indicated that the pH of the solution was the largest contributing factor (94.41%) with statistical significance. Models of experimental *versus* predicted *K*/*S* values indicated that these values were strongly connected with an *R*^2^ of 89.0% and Pearson coefficient (*P* value was 0.000). The *E*%, *F*%, and *T*% values of the grass cloth dyeing under the optimised conditions in palm oil media were 95.41%, 50.61%, and 48.30%, respectively, and the colourfastness to washing and rubbing was satisfactory. The XRD analysis confirmed the cellulose I pattern, and the dyeing process did not affect the crystallinity of the grass cloth fibre. Finally, the palm oil recyclability was around 99%, and the water consumption was less than that required for conventional water-based dyeing, which was undoubtedly a promising gateway for the development of a palm oil-based sustainable textile dyeing system.

## Author contributions

L. L., L. X., L. L., C. Z., Y. C.: concept, data analysis, writing – original draft. L. L., L. X., Y. C., M. N. P., M. S. I., Y. Z., and V. N.: writing – review & editing. Y. C. and M. M. H.: conceptualization, funding acquisition, project administration, supervision, writing – review & editing.

## Conflicts of interest

There are no conflicts to declare.

## Supplementary Material
